# Recurrent Thyrotoxic Periodic Paralysis Precipitated by Alcohol: A Case Report of Diagnostic Delay in a Young Male With Graves' Disease

**DOI:** 10.7759/cureus.112561

**Published:** 2026-07-13

**Authors:** Makhlouf Bannoud, Lucia Hong, Daniel Woo, Zachary R Daniels, Ali Alkatifi

**Affiliations:** 1 Internal Medicine, University of California, Riverside School of Medicine, Riverside, USA

**Keywords:** acute management of thyrotoxicosis, alcohol use, graves' disease, hyperthyroidism, thyrotoxic hypokalemic periodic paralysis

## Abstract

Thyrotoxic periodic paralysis (TPP) is a rare complication of hyperthyroidism characterized by acute weakness and hypokalemia due to intracellular potassium shift. Clinical management of TPP consists of cautious potassium replacement, non-selective β-blockade, and prompt treatment of the underlying thyrotoxicosis. Definitive control of hyperthyroidism, combined with avoidance of known precipitants, is essential to prevent recurrent attacks. Failure to recognize the syndrome can result in repeated admissions, unnecessary evaluations, and life-threatening complications. We report the case of a 30-year-old Samoan man with Graves’ disease who experienced recurrent episodes of paralysis over several months before the correct diagnosis was established. His initial presentations with severe hypokalemia and weakness were attributed to gastrointestinal and spinal disease, leading to repeated misdiagnoses despite multiple admissions. Alcohol intake, a less common but clinically important trigger, frequently precipitated his episodes; despite counseling, he was unable to achieve complete abstinence. He ultimately developed paralysis complicated by cardiac arrest, after which thyroid testing confirmed Graves’ disease and TPP. Despite medical therapy, relapses continued due to lack of definitive thyroid treatment, intermittent methimazole lapses, ongoing alcohol use, and barriers to specialty follow-up. This case underscores the diagnostic challenges of TPP, the importance of recognizing it in Asian and Latino populations where prevalence is higher, and the need for early thyroid evaluation in patients with recurrent hypokalemic paralysis to prevent fatal outcomes.

## Introduction

Thyrotoxic periodic paralysis (TPP) is an acquired form of hypokalemic periodic paralysis that occurs in the setting of thyrotoxicosis, most often Graves’ disease. It is more commonly reported in Asian and Latino men [1] but remains underrecognized in Western practice. The condition is characterized by acute, reversible episodes of flaccid paralysis associated with hypokalemia, hypophosphatemia, and hypomagnesemia due to intracellular electrolyte shifts.

Well-recognized precipitants include carbohydrate-rich meals, strenuous exercise, and emotional stress. Alcohol intake, although less commonly reported, is an important and clinically relevant trigger that may complicate both diagnosis and management [2]. Delayed recognition can result in repeated hospitalizations, misattribution to gastrointestinal or neurologic causes, and life-threatening complications such as arrhythmias and respiratory failure [1].

We report the case of a 30-year-old Samoan man with Graves’ disease who experienced recurrent episodes of paralysis, frequently precipitated by alcohol, over the course of a year before his thyroid status was evaluated. His course was complicated by cardiac arrest, ongoing relapses despite therapy, and barriers to definitive thyroid treatment. Psychiatry and social work were also involved to address comorbid depression and psychosocial stressors, underscoring the multidimensional challenges of managing TPP.

## Case presentation

A 30-year-old Samoan man with a history of peptic ulcer disease, alcohol and cocaine use disorder, a family history of Graves' disease, and hypertension presented on day 0 with acute bilateral lower extremity weakness and numbness upon awakening. He described heaviness, cramping, and inability to ambulate. Similar episodes had occurred approximately a dozen times over the prior year, typically in the early morning and resolving within one to two hours.

His first documented episode was approximately 10 months before day 0, when he presented with bilateral leg weakness and severe hypokalemia (serum potassium 2.1 mmol/L). An MRI of the lumbar spine showed mild lumbar spondylosis without central canal stenosis and varying degrees of bilateral foraminal narrowing. The episode was attributed to gastrointestinal losses from chronic diarrhea and to the previously noted lumbar spondylosis. Thyroid function was not obtained, and he was discharged with electrolyte replacement.

Approximately six months before day 0, he was readmitted with profound hypokalemia, muscle weakness, and shallow breathing. During this admission, he developed cardiac arrest requiring resuscitation and intensive care. A broader evaluation finally revealed an undetectable TSH with elevated free T₃ and free T₄, and antibody testing, including elevated TSH receptor antibodies (Table 1), confirmed Graves’ disease. He was diagnosed with TPP, and after he was stabilized, he was discharged on methimazole, propranolol, and oral potassium.

**Table 1:** Admission laboratory values demonstrating elevation of TgAb, TPOAb, and TRAb TgAb: thyroglobulin antibodies, TPOAb: thyroid peroxidase antibodies, TRAb: thyroid-stimulating hormone receptor antibodies

Notable lab results	Results on presentation	Normal reference range
TgAb	23.8 IU/mL	<4 IU/mL
TPOAb	456.4 IU/mL	<5.6 IU/mL
TRAb	Elevated	<1.75 IU/L

**Figure 1:**
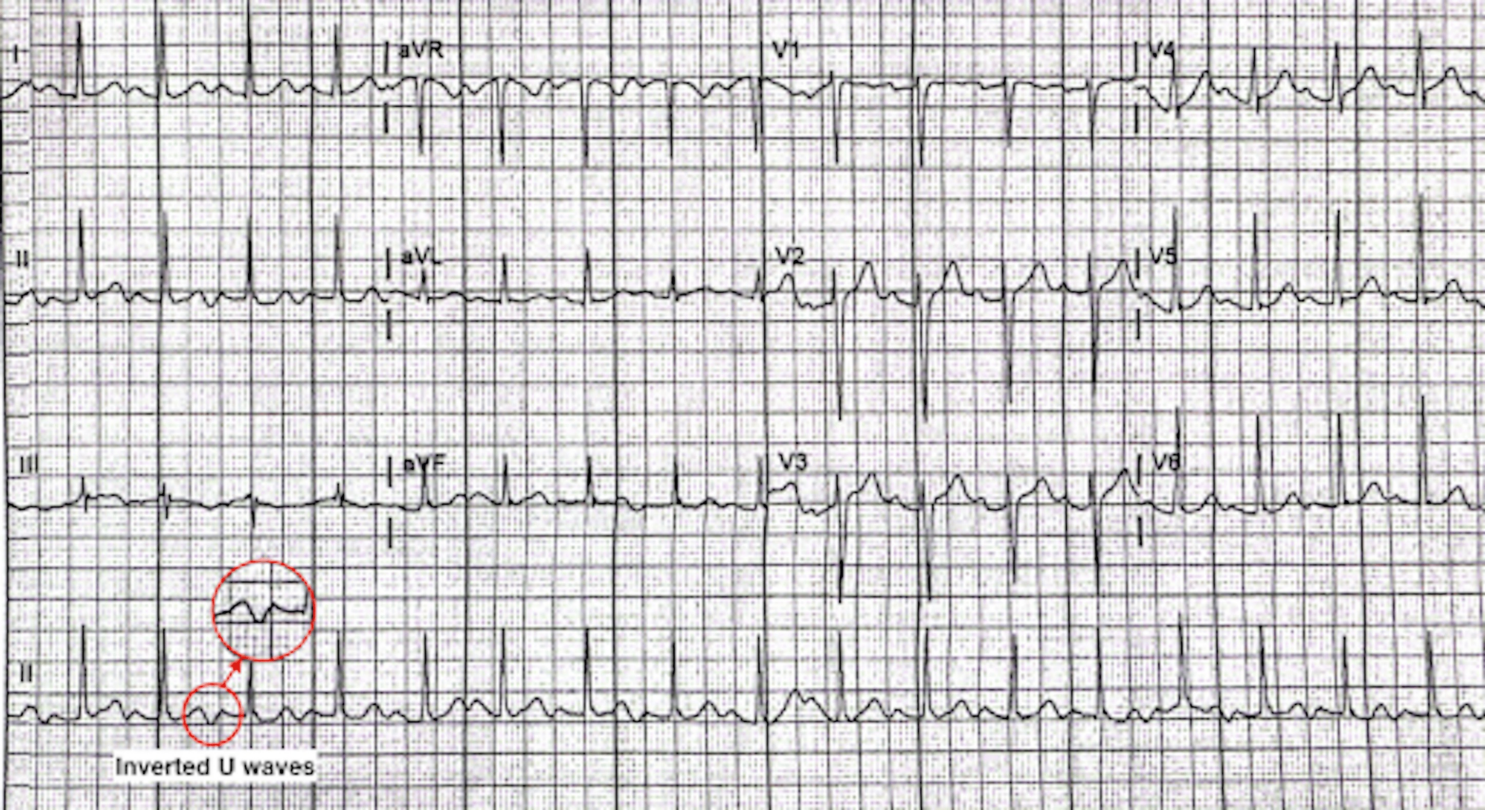
Electrocardiogram demonstrating inverted U waves in lead II, a classic but uncommon manifestation of hypokalemia

Despite adherence to therapy, he reported frequent relapses in the following months, most often in the early morning and occasionally after alcohol intake. Approximately four months before day 0, alcohol ingestion at a family event precipitated complete paralysis of the lower extremities. Although he attempted to reduce his alcohol use afterward, he did not achieve full abstinence. On day 0, after a three-day lapse in methimazole, he again presented with bilateral leg weakness and hypokalemia. On arrival, vital signs were within normal physiologic ranges; the neurologic exam revealed profound lower-extremity weakness with intact cranial nerves. Laboratory studies showed severe hypokalemia, hypomagnesemia, and suppressed TSH with elevated thyroid hormone levels (Table 2). ECG demonstrated sinus tachycardia with inverted U waves in lead II (Figure 1), and toxicology was positive for cocaine, which may have contributed to electrolyte shifts but did not fully explain his recurrent episodes.

**Table 2:** Admission laboratory values demonstrating severe hypokalemia, hypomagnesemia, hypophosphatemia, and thyrotoxicosis FT3: free triiodothyronine, FT4: free thyroxine, TSH: thyroid-stimulating hormone

Notable lab results	Results on presentation	Normal reference range
Sodium	140 mmol/L	135-145 mmol/L
Potassium	2 mmol/L	3.5-5.0 mmol/L
Magnesium	1.3 mg/dL	1.7-2.2 mg/dL
Phosphorus	1.2 mg/dL	2.5-4.5 mg/dL
FT3	8.1 pg/mL	2.0-4.4 pg/mL
FT4	3.09 ng/dL	0.8-1.8 ng/dL
TSH	Nondetectable	0.4-4.0 µIU/mL

The patient was admitted to telemetry, where initial treatment consisted of 80 mEq of intravenous potassium chloride administered over five hours and 4 g of intravenous magnesium sulfate administered over four hours, with continuous telemetry monitoring. Methimazole 20 mg orally once daily and propranolol 3 mg/kg/day orally were initiated, and serum electrolytes were rechecked approximately two hours after initiation of electrolyte replacement and serially thereafter until potassium and magnesium levels normalized, with corresponding resolution of muscle weakness. Psychiatry was consulted for comorbid depression (PHQ-9 = 15) [3], and social work was engaged given recent marital separation and barriers to specialty follow-up. He was discharged with outpatient endocrinology follow-up for definitive thyroid therapy, which had not yet been initiated.

## Discussion

TPP is a rare but serious complication of thyrotoxicosis, most often associated with Graves’ disease. It is characterized by acute episodes of flaccid muscle weakness and hypokalemia, which resolve with normalization of potassium levels. The pathogenesis centers on an exaggerated intracellular shift of potassium into skeletal muscle. Thyroid hormone enhances Na⁺/K⁺-ATPase activity both genomically, by upregulating transcription through thyroid hormone response elements, and nongenomically, by increasing pump activity and membrane insertion; these effects are amplified by β-adrenergic stimulation and hyperinsulinemia [1]. Skeletal muscle potassium channels, particularly inward rectifier (Kir) channels, also play a critical role in maintaining extracellular potassium balance. In TPP, reduced outward potassium efflux through Kir channels, whether due to functional inhibition or genetic variants such as Kir2.6 (KCNJ18) mutations reported in up to ~33% of cases, contributes to “trapping” of potassium within myocytes. The combined result is paradoxical depolarization, sodium channel inactivation, and subsequent muscle inexcitability and paralysis [1,2].

Concomitant electrolyte abnormalities, such as hypophosphatemia and hypomagnesemia, may amplify neuromuscular dysfunction and increase arrhythmic risk. Episodes of TPP are typically precipitated by factors that promote adrenergic or insulin surges. These include carbohydrate-rich meals, strenuous exercise, emotional stress, trauma, infection, and medications such as insulin and diuretics [4]. Alcohol ingestion, although less commonly reported, is a clinically important precipitant due to its effects on insulin secretion, adrenergic tone, and magnesium balance. In our patient, alcohol consistently preceded paralysis episodes and represented a key driver of relapse despite efforts at reduction. Cocaine use may also have contributed to adrenergic overstimulation and electrolyte shifts, though its role remains less well established. Attacks classically arise at night or in the early morning, often a few hours after a heavy meal, alcohol intake, or strenuous exercise, with complete recovery typically occurring within 72 hours [1,5].

In addition to acting as an acute precipitant, chronic alcohol use may further complicate the recognition of TPP. A recent case report from Taiwan described a 41-year-old man with Graves’ disease and long-standing alcohol abuse who presented with profound hypokalemia (<1.5 mEq/L) but without classic thyrotoxic or gastrointestinal symptoms. The authors suggested that chronic alcohol consumption may not only predispose to hypokalemia through gastrointestinal losses, hypomagnesemia, and renal tubular dysfunction, but also blunt the perception of symptoms, thereby delaying diagnosis and treatment. This case reinforces that alcohol can both trigger attacks and mask their severity, increasing the risk of delayed recognition and misdiagnosis in patients with hyperthyroidism [6].

The diagnosis of TPP can be challenging, as symptoms of thyrotoxicosis are often subtle or absent, and initial presentations may mimic other causes of hypokalemic paralysis. Our patient’s recurrent episodes were initially attributed to gastrointestinal losses and spinal pathology, leading to repeated hospitalizations and a delay in thyroid testing. This diagnostic delay culminated in a life-threatening cardiac arrest. Similar pitfalls have been reported in a Qatari cohort of 18 patients, where 83% had no prior history of hyperthyroidism and 50% experienced multiple attacks before diagnosis; moreover, only two-thirds demonstrated obvious clinical signs of thyrotoxicosis on presentation [7]. Consistent with these observations, a long-term cohort found that most patients were first labeled with periodic paralysis rather than thyrotoxicosis (24/33), with a median delay of approximately five months (range one week to 12 years) to the correct diagnosis; attacks were predominantly nocturnal/early morning and often followed carbohydrate loads or rest after exercise. Notably, potassium may normalize by the time blood is drawn, thereby obscuring recognition [1]. These data, together with our case, underscore the importance of considering TPP in any patient with recurrent hypokalemic paralysis, particularly in Asian or Latino men, among whom prevalence is higher [5,7], and of obtaining thyroid function testing early to distinguish TPP from familial hypokalemic periodic paralysis, neuromuscular disorders, or other metabolic derangements.

Acute management requires cautious potassium replacement, as total body potassium is not depleted and aggressive repletion can lead to rebound hyperkalemia once the intracellular shift resolves. Rebound hyperkalemia has been reported in up to ~42% of episodes, particularly when high-dose replacement is given. For this reason, potassium is best administered orally or intravenously at a controlled rate (≈25-50 mmol over approximately two hours) and discontinued once muscle strength begins to recover [1]. Although some have proposed prophylactic potassium to prevent recurrences, evidence supporting this approach is inconsistent and not routinely recommended; potassium supplementation should be reserved for acute attacks when hypokalemia is present [8].

Non-selective β-blockers such as propranolol (80-160 mg/day) rapidly reverse paralysis by blocking adrenergic stimulation of Na⁺/K⁺-ATPase activity and can be used as first-line therapy, either alone or with cautious potassium supplementation, without provoking rebound hyperkalemia [9-11]. Definitive therapy requires prompt treatment of the underlying thyrotoxicosis, most often radioactive iodine ablation or thyroidectomy, as attacks cease when euthyroidism is achieved; adherence barriers and precipitating behaviors (e.g., alcohol use) should be addressed concurrently to prevent relapse [1]. Several alternative agents have been explored for prevention of attacks, but with variable and sometimes contradictory results. Acetazolamide, commonly used in familial periodic paralysis, has produced mixed outcomes in TPP, ranging from improvement in some series to induction or worsening of paralysis in others, making it unreliable and potentially harmful [8,12].

Moreover, this case illustrates the importance of multidisciplinary care in managing TPP. The involvement of psychiatry and social work was essential for addressing comorbid depression, substance use, and social instability, all of which had a significant impact on treatment adherence and patient outcomes. Without addressing these factors, medical therapy alone would have been insufficient in preventing relapse and achieving optimal recovery. Incorporating a comprehensive care plan that includes psychosocial support is crucial in managing the complexities of TPP.

## Conclusions

TPP should be considered in any patient with recurrent episodes of hypokalemic paralysis, particularly in at-risk populations, even in the absence of overt thyrotoxic symptoms. Alcohol use should be recognized as a clinically relevant trigger, and lifestyle modifications, including reducing alcohol consumption, should be emphasized alongside medical therapy. Early thyroid testing, cautious potassium replacement, and prompt initiation of definitive thyroid treatment are critical to preventing recurrence and potentially fatal complications. Additionally, a multidisciplinary approach, involving psychiatry, social work, and addiction support, is essential for improving treatment adherence and long-term outcomes for patients with TPP.
